# Impact of air pollution on life expectancy: the role of digitalization, urbanization and institutional quality in BRICS economies

**DOI:** 10.3389/fpubh.2026.1681381

**Published:** 2026-04-24

**Authors:** Hong Zhang, Haixiang Qiao, Xiao Gu, Ijaz Uddin, Xiuping Li, Zhenhua He, Xiaolan Zhang

**Affiliations:** 1 School of Business, Jinggangshan University, Ji’an, China; 2 School of Finance and Economics, Qingdao Binhai University, Qingdao, China; 3 Urban and Rural Community Governance Modernization Research Center (HOU), Hangzhou, China; 4 Department of Economics, Abdul Wali Khan University Mardan, Mardan, KPK, Pakistan; 5 Shaoxing Institute of Technology, Shaoxing, China; 6 The First Affiliated Hospital of Zhejiang Chinese Medical University (Zhejiang Provincial Hospital of Chinese Medicine), Zhejiang, Hangzhou, China

**Keywords:** air pollution, BRICS, digitalization, institutional quality, life expectancy, urbanization

## Abstract

Air pollution lowers life expectancy (LEX) by increasing the risk of respiratory, cardiovascular, and other chronic diseases. However, digitalization can help monitor air quality and support public health awareness through real-time data. Institutional quality ensures effective enforcement of environmental regulations and pollution control policies. Together, they help reduce pollution exposure and protect public health. Therefore, the current study aims to analyze the impact of Air pollution on LEX, with the role of urbanization, digitalization, and institutional quality in BRICS economies. This study employs the Cross-Sectionally Augmented Autoregressive Distributed Lag (CS-ARDL) model, the Fixed Effects estimator, and the Fully Modified Ordinary Least Squares (FMOLS) technique. The findings indicate that CO_2_ emissions have a negative impact on LEX, whereas GDP, urbanization, digitalization, and institutional quality have a positive effect on LEX. This study recommended that policymakers focus on reducing CO_2_ emissions by promoting clean energy and enforcing environmental regulations to mitigate their harmful effects on life expectancy. It also emphasized the importance of enhancing urban infrastructure to improve access to healthcare and sanitation. Additionally, the study highlighted the role of digitalization in strengthening health awareness and service delivery, and the need to improve institutional quality for effective health and environmental governance.

## 1 Introduction

Life expectancy (LEX) is a key indicator of population health and is directly aligned with Sustainable Development Goal 3 (SDG 3: Good Health and Wellbeing), which aims to ensure healthy lives and promote wellbeing for all at all ages (1, 2). Air pollution is widely recognized as a significant public health hazard (3–7). CO_2_ emissions (CO_2_e) are a major contributor to environmental degradation (8–10). Globally, CO_2_e from fossil fuel combustion have generated substantial negative externalities. The environment has been adversely affected by energy consumption particularly fossil fuels alongside manufacturing and construction activities. These processes have led to poor health outcomes, reduced LEX, and widespread environmental degradation, including harm to aquatic ecosystems (11–14). The World Health Organization estimates that ambient air pollution caused approximately 4.2 million premature deaths worldwide in 2016. Given that nearly 90% of the global population resides in areas with hazardous air quality, this burden is expected to increase further (15, 16). Environmental deterioration can negatively affect population health through multiple pathways. Severe outdoor air pollution contributes to increased premature mortality and the prevalence of chronic diseases such as lung cancer, cardiovascular disease, and asthma (17–19).

Information and communication technology (ICT) has both direct and indirect effects on LEX. Specifically, ICT can enhance LEX by facilitating the sharing of information and improving access to knowledge related to health, disease outbreaks, and healthy diets (20). ICT has the potential to transform public health by influencing both economic and social dimensions. High-income countries can invest in medical information and communication technologies to reduce child mortality and improve maternal health outcomes (21). In developing countries, prioritizing medical information technology projects may yield rapid benefits, particularly when economic downturns constrain the implementation of comprehensive online health policies (22, 23). Digital technologies also create new opportunities for health education and public awareness initiatives. Social media platforms, websites, and mobile applications enable the rapid dissemination of evidence-based health information to broad and diverse populations. Through these channels, digitalization supports disease prevention, promotes healthy lifestyles, and facilitates early disease detection (24). Moreover, ICT provides platforms for health-oriented technological solutions, including electronic medical record systems, health data monitoring, short message service (SMS) interventions, diagnostic support, and remote treatment through telemedicine. These technologies enhance the distribution, management, tracking, and diagnosis of health information (25). Qiang et al. (26) emphasize the role of ICT particularly mobile health (mHealth) in improving therapeutic support, patient monitoring, supply chain management, health financing, and emergency response services. ICT tools also help reduce delays in drug transportation and offer point-of-use technologies. In addition, virtual healthcare increasingly substitutes in-person consultations with telephone- or video-based services, expanding access to healthcare delivery (27).

The conversion of rural areas into urban areas, along with migration to locations offering improved socioeconomic and health conditions such as better employment opportunities, access to healthcare, and education are the primary drivers of urban population growth (28, 29). Urbanization (URB) refers to the expansion and development of human settlements characterized by built environments, including housing, transportation networks, and public infrastructure. This process is commonly associated with socioeconomic progress, as it generally improves access to education, employment, sanitation, and healthcare services. Urban areas also provide a wide range of cultural and recreational amenities, such as parks, gardens, playgrounds, and sports facilities, which contribute to better mental and physical wellbeing. Residents of urban centers typically earn higher incomes, enabling greater investment in health-related goods and services. Moreover, the expansion of urban areas has improved access to modern and technologically advanced medical care, thereby contributing to increased life expectancy (30). There are two prevailing perspectives regarding the impact of urbanization on life expectancy. First, URB contributes to improvements in LEX by enhancing access to essential services, including healthcare, clean drinking water, and sanitation systems. Urban regions are generally equipped with more advanced medical facilities, preventive healthcare programs, and efficient emergency response systems. Additionally, urban areas promote higher educational attainment, which is closely associated with healthier behaviors and greater awareness of health-related issues. The wider range of economic opportunities available in urban settings often leads to higher income levels, improved housing conditions, adequate nutrition, and an enhanced overall quality of life factors that collectively reduce mortality rates and support longer life spans (30). Second, URB can exert negative effects on human health, the environment, and social behavior. By 2050, it is projected that 68% of the global population will reside in urban areas, up from 55% in 2018 (31, 32). URB is associated with various mental and physical health challenges. Individuals living in cities are more likely to be exposed to risk factors for non-communicable diseases, including depression, diabetes, and cardiovascular diseases (29, 33). Moreover, evidence from the COVID-19 pandemic indicates that the size of the urban population was directly correlated with the number of confirmed cases, suggesting that higher population density in urban areas increases the risk of infectious disease transmission (34).

This study examines the impact of air pollution on LEX, with particular emphasis on the roles of digitalization, urbanization, and institutional quality in BRICS economies. While prior studies have extensively explored the environmental and socio-economic determinants of life expectancy, they have largely overlooked the combined roles of digitalization, urbanization, and institutional quality, especially within the BRICS context. Recent developments—such as rapid digital transformation, accelerated urban expansion, evolving institutional structures, and increasing levels of air pollution particularly in BRICS countries, therefore call for a renewed empirical investigation. The primary contribution of this study lies in its integrated analysis of digitalization and urbanization within a health outcomes framework, an area that remains underexplored in the existing literature. Second, the study jointly examines environmental, digital, social, and institutional factors to provide a more comprehensive understanding of the determinants of life expectancy in BRICS economies. Third, the findings offer important policy implications to support health-oriented, environmentally sustainable, and institutionally sound development strategies in emerging economies.

The remainder of the study is organized as follows: Section 2 reviews the relevant literature on the topic. Section 3 outlines the data sources and describes the empirical methodology. Section 4 presents the empirical results along with a detailed discussion. Finally, Section 5 concludes the study by summarizing the key findings.

## 2 Literature review

### 2.1 Carbon emissions and life expectancy nexus

A substantial number of studies reported a negative association between CO_2_ emissions (CO_2_e) and life expectancy (LEX). For example, Osabohien et al. (14), using the ARDL model for Nigeria (1980–2017), found that CO_2_e significantly reduced LEX by approximately 0.35%, emphasizing the need to transition toward low-carbon energy sources. Similarly, Das and Debnath (9), applying the ARDL bounds testing approach to India (1991–2018), identified a long-run quadratic relationship and concluded that India had exceeded its optimal CO_2_e threshold, reinforcing the urgency of emission reduction strategies. Evidence from a broader regional perspective also supported this negative linkage. Azam and Adeleye (35), employing bootstrap OLS and system GMM for 36 Asia-Pacific countries (2005–2010), demonstrated that emissions from both liquid and solid fuels adversely affected LEX, with liquid fuels exerting the strongest impact. Likewise, Shaari et al. (36), using FMOLS, DOLS, and CS-ARDL for ASEAN-5 countries (1995–2020), confirmed that CO_2_e consistently reduced LEX, while green technology and health expenditure improved it. Redzwan and Ramli (31), in the case of Malaysia (1997–2021), found significant short-run effects of CO_2_e on LEX, highlighting the importance of immediate policy responses. Beyond the direct pollution–health nexus, several studies integrated moderating and complementary factors. Ibrahim (37), applying second-generation panel techniques including CS-ARDL and quantile regression for oil-abundant African countries (1980–2019), showed that fossil fuels and carbon emissions reduced LEX, but income growth and human capital investment could mitigate these adverse effects. Guo et al. (38), analyzing SAARC countries (1990–2022) through panel ARDL and quantile regression, further confirmed that CO_2_ emissions reduced LEX and increased infant mortality, whereas renewable energy and URB could improve health outcomes when properly managed. Osei-Kusi et al. (39), in a comparative study across Sub-Saharan Africa, MENA, and Europe and Central Asia using panel data for 82 countries over 30 years, reported a positive relationship between CO_2_e, energy use, and LEX in certain regions, while also identifying a negative association between CO_2_e and mortality in some cases.

### 2.2 Digitalization and life expectancy nexus

Several studies identified a direct and statistically significant positive effect of ICT on LEX. Ronaghi (40), analyzing Middle Eastern countries over the period 2008–2018 using panel data techniques and the Hausman test, found that ICT exerted a strong positive impact on LEX, with a regression coefficient of 0.551. Similarly, Majeed and Khan (41), employing fixed effects, two-stage least squares, and system GMM methods across 184 countries (1990–2014), demonstrated that internet usage, mobile subscriptions, and fixed telephone lines significantly enhanced LEX. These findings suggested that improvements in ICT infrastructure strengthened health systems, facilitated access to medical information, and improved service delivery. Beyond direct effects, other studies explored causal and mediating mechanisms. Bayar et al. (42), using symmetric and asymmetric causality tests for emerging market economies (1997–2020), identified a bidirectional causal relationship between mobile subscriptions and LEX, alongside a unidirectional causal link from internet usage to LEX in several countries. This evidence implied that ICT not only influenced health outcomes but also expanded as societies experienced improvements in longevity and human development. In a similar vein, Shao et al. (22), applying fixed effects and Sobel mediation tests to panel data from 141 countries (2012–2016), confirmed that ICT partially mediated national health outcomes, reinforcing the idea that digital technologies operated through indirect channels such as education, income, and institutional efficiency. More recent contributions emphasized spatial and structural heterogeneity. Pu et al. (43), using a Geographically and Temporally Weighted Regression (GTWR) model across 182 countries (1990–2020), found a significant positive impact of internet usage on LEX, with considerable regional variation. This highlighted that the health benefits of ICT were not uniform but depended on socioeconomic conditions, technological diffusion, and governance structures. Likewise, Alhassan and Adam (44), employing PLS-SEM for 121 countries (2018), showed that digital inclusion and ICT access significantly improved quality of life, indirectly supporting health indicators such as LEX.

### 2.3 Urbanization and life expectancy nexus

Ahmad et al. (45), using a random effects model for six South Asian countries (1997–2021), found that URB and income inequality both reduced LEX for males and females, although health expenditure mitigated these adverse effects. Similarly, Amin et al. (46), applying FMOLS to ASEAN-5 countries (1995–2020), reported that while economic growth and health expenditure improved LEX, urbanization exerted a significant negative impact, underscoring the need for structured urban planning. Historical evidence also supported the notion of an “urban penalty.” Torres et al. (47), examining Scotland (1861–1910), showed that rapid urban population shifts lowered overall LEX due to elevated mortality in densely populated cities. Erum et al. (48), focusing on highly polluted countries (1990–2022) using MMQR and panel techniques, further confirmed that URB and poor air quality jointly reduced LEX, particularly where governance and environmental regulations were weak. Conversely, other studies emphasized the potential health gains associated with well-managed urbanization. Panahi and Aleemran (49), analyzing MENA countries (2000–2012), found that URB and health expenditure positively influenced LEX, while inflation had a detrimental effect. Zhang et al. (30), employing ARDL and FMOLS for China (1990–2022), concluded that urbanization, education, and green growth enhanced LEX, although CO_2_e offset part of these gains. Wang et al. (50) similarly reported positive short- and long-run effects of URB on global health, noting reductions in mortality and modest improvements in LEX, but cautioned that air pollution weakened these benefits, especially in lower-income regions. Guo et al. (38), studying SAARC countries (1990–2022), also found that URB, alongside renewable energy, GDP, and industrialization, positively contributed to LEX, while CO_2_e consistently reduced it.

### 2.4 Institutional quality and life expectancy nexus

Several studies emphasized the moderating role of income and institutional frameworks in the energy–health nexus. Ibrahim and Ajide (51), focusing on four oil-producing African countries (1990–2017), employed panel unit root and cointegration tests alongside FMOLS estimation. Their findings indicated that non-renewable energy consumption reduced LEX. While higher income levels mitigated this adverse effect, institutional quality unexpectedly exacerbated it, suggesting that governance structures in resource-dependent economies did not effectively regulate environmental externalities. In contrast, more recent cross-country analyses generally reported a positive role of institutional quality in improving health outcomes. Uddin et al. (52) examined six South Asian countries (2002–2020) using CS-ARDL, FMOLS, and DOLS and found that IQ, health expenditure, and financial development significantly increased LEX, whereas CO_2_ emissions, ecological footprint, and population growth reduced it. Similarly, Hadipour et al. (53), analyzing 158 countries (2001–2020) and constructing governance indices through PCA, demonstrated that IQ, GDP, schooling, and urbanization enhanced LEX, while CO_2_ emissions weakened it. These findings underscored the importance of effective institutions in facilitating environmental regulation, efficient public spending, and improved healthcare delivery. Evidence from regional studies further reinforced the positive institutional effect. Vatamanu et al. (54), using FMOLS and panel cointegration techniques for EU countries (2000–2020), found that both renewable energy and IQ significantly improved LEX. Nica et al. (55), employing CS-ARDL and quantile regression for Eastern European countries (1990–2021), similarly concluded that IQ, health expenditure, and renewable energy raised LEX, whereas fossil fuels and CO_2_ emissions reduced it. These results suggested that institutional capacity strengthened the benefits of clean energy transitions and public health investments. Beyond environmental and energy considerations, institutions also buffered external economic shocks. Ha and Nam (56), applying structural gravity models to 148 countries (1995–2018), found that global economic sanctions negatively affected LEX, particularly in developing nations. However, stronger institutional quality and financial openness mitigated these harmful effects, highlighting governance as a resilience mechanism in times of crisis. Complementing this perspective, Zhang et al. (30), using machine learning techniques for high-longevity countries, identified rule of law and government effectiveness as key determinants that positively influenced LEX.

### 2.5 Theoretical review

According to Zhang et al. (30), Urbanization influences LEX in both beneficial and adverse ways. On one hand, it often enhances access to healthcare, education, and improved living conditions. On the other hand, poorly planned urban growth can lead to environmental deterioration, overburdened health systems, and increased exposure to lifestyle-related diseases, which may negatively affect life expectancy. The rapid and often unregulated urban expansion seen in the 21st century has significantly altered both natural ecosystems and built environments, with serious consequences for environmental quality and human health. This highlights the importance of social structures in shaping sustainable urban societies. Notably, Asia and Africa are home to the world’s fastest-growing urban centers, positioning urban life as the dominant living arrangement for both present and future generations, with far-reaching implications for global health, culture, and societal development.

According to Bayar et al. (42), ICT can influence life expectancy through several direct and indirect channels. On the positive side, greater access to ICT improves the availability and sharing of health-related information, including guidance on disease prevention, healthy nutrition, and epidemic awareness. Online health resources enhance individuals’ health knowledge, strengthen doctor–patient communication, and support early diagnosis and timely treatment of diseases. ICT also improves the efficiency of healthcare systems by enabling better use of clinical time and resources, which contributes to improved health outcomes and quality of life. Excessive or unbalanced ICT use may adversely affect life expectancy. Reduced physical activity associated with high ICT penetration can increase the risk of obesity, cardiovascular diseases, and musculoskeletal disorders. Prolonged screen time is also linked to health issues such as back and neck pain, joint and muscle problems, eye and hearing disorders, and overall physical inactivity, which can negatively affect long-term health. Beyond health-related channels, ICT influences life expectancy indirectly through broader economic and environmental pathways. It shapes economic growth, financial development, employment conditions, innovation, entrepreneurship, energy use, green energy development, and electronic waste generation, all of which have implications for population health and longevity (42).

There is broad agreement that institutional quality is a key determinant of health outcomes. Strong institutions create an environment that supports cooperative solutions and fosters better economic performance. Evidence also suggests that many middle-income countries have struggled to catch up with developed nations despite having sound policies, mainly due to weak institutional frameworks (57). While some studies argue that human capital may play a more critical role than institutions in driving growth, good institutions still contribute to health by shaping universal policies, such as access to high-quality healthcare, health insurance, and public health programs. Moreover, effective institutions provide guidance and information on hygiene, healthy practices, and other knowledge that benefits the general population, particularly the most vulnerable. Overall, these factors suggest that improvements in environmental and health outcomes are closely linked to institutional quality (57). Uddin et al. (52) estimated the extended version of the health production function proposed by Grossman (58) incorporating the environmental, institutional factors. And found that ecological degradation has an adverse effect on LEX, while the institutional quality has a positive impact on LEX.

## 3 Model, methodology and data

### 3.1 Model

To examine the impact of CO_2_ emissions, ICT, urbanization, and IQ on life expectancy in BRICS countries, the empirical model of this study is developed based on previous literature. This model captures the relationship between environmental, technological, socio-economic, and governance factors and life expectancy, as identified in earlier empirical studies. The functional form of the model is specified as follows:


$$\begin{array}{l} {\mathrm{LEX}}_{,\mathrm{it}}=\beta_{0}+\beta_{1}\mathrm{CO}2e_{\mathrm{it}}+\beta_{2}{\mathrm{ICT}}_{\mathrm{it}}+\beta_{3}{\mathrm{URB}}_{\mathrm{it}}\\ +\beta_{4}{\mathrm{IQ}}_{\mathrm{it}}+\beta_{5}{\mathrm{GDP}}_{\mathrm{it}}+e_{i,t} \end{array}$$ (1)

Where in Equation 1, *LEX, CO_2_e, ICT, URB, IQ*, and *GDP* represent life expectancy, carbon dioxide emissions, information and communication technology, urbanization, institutional quality, and gross domestic product, respectively. The subscripts *i* and *t* denote the country and time period, while $e_{i,t}$ refers to the error term. In the model, LEX is the dependent variable, whereas *CO_2_e, ICT, URB, IQ*, and *GDP* serve as independent variables. $\beta_{0}$ represents the intercept, and $\beta_{1}\;\text{to}\;\beta_{5}$ are the slope coefficients. To reduce data skewness and heteroscedasticity, all variables LEX, CO_2_e, URB, and GDP are transformed into natural logarithms. However, IQ and ICT are left in their original form due to the presence of negative values.

### 3.2 Methodology

In econometric analysis, preliminary tests are conducted on the data series before selecting an appropriate estimator. Since LEX or other variables in one country may be influenced by those in another, it is important to account for potential cross-sectional dependence (CSD). To address this, the study applies the CD tests and the Lagrange multiplier (LM) test. This test is used to detect cross-sectional interdependence among panel units. The LM and CD test equations are presented in Equations 2, 3, as follows:


$$\mathrm{LM}=J\sum_{i=1}^{K-1}\sum_{j=i+1}^{K}{{\hat{\propto }}^{2}}_{\mathrm{ij}}$$ (2)

$$\mathrm{CD}=\sqrt{\frac{2K}{N(N-1)}}[\sum_{i=1}^{K-1}\sum_{j=i+1}^{K}{{\hat{\varphi }}^{2}}_{\mathrm{ij}}]$$ (3)

Where ${{\hat{\varphi }}^{2}}_{\mathrm{ij}}$ represent the residuals correlation between nation *i* and nation *j*, respectively [see Uddin et al. (52)].

In the second stage of the pre-tests, the stationarity of the series was examined. This study utilized the first generation (LLC) and second generation (CIPS). Given the presence of CSD in the data, the study employed the CIPS unit root test proposed by Pesaran (59), which belongs to the second generation of unit root tests. Unlike first-generation tests, the CIPS test accounts for cross-sectional dependence, making it more effective and reliable in such settings. Therefore, it provides more robust and consistent results when applied to panels with interdependent units. The CIPS unit root test is formulated as shown in Equation 4.


$$\Delta Y_{\mathrm{it}}=\omega_{i}+\omega_{i}Y_{\mathrm{it}-1}+\omega_{i}{\bar{Z}}_{t-1}+\sum_{i=0}^{p}\omega_{\mathrm{iI}}\Delta {\bar{Y}}_{t-1}+\sum_{i=1}^{p}\omega_{\mathrm{iI}}\Delta Z_{\mathrm{it}-I}+e_{\mathrm{it}}$$ (4)

$$\hat{\text{CIPS}}=\frac{1}{K}\sum_{i=1}^{n}\mathrm{CAD}F_{i}$$ (5)

In Equation 4, ${\bar{Z}}_{t-1}$ and $\Delta {\bar{Y}}_{t-1}$ link to the cross-sectional average. Equation 5, shows the CIPS test.

After testing the unit root test, this study utilizes the Kao residual cointegration test (60) is a panel cointegration test based on Engle-Granger methodology. It tests for the presence of a long-run equilibrium relationship among panel variables using residual-based methods. A significant test statistic (e.g., ADF t-statistic) leads to rejection of the null hypothesis of no cointegration. The Johansen Fisher Panel Cointegration Test (61) combines individual Johansen tests using Fisher’s approach. It considers both the Trace and Max-Eigen statistics to determine the number of cointegrating vectors across panel units. This test allows for heterogeneity in cointegrating relationships across countries or units in the panel.

In this study, we employ the Cross-Sectional Autoregressive Distributed Lag (CS-ARDL) model, which is suitable when panel variables exhibit mixed orders of integration, i.e., some are stationary at level [*I*(0)] and others at first difference [*I*(1)]. This flexibility allows the CS-ARDL model to estimate both short-run and long-run relationships without requiring all variables to be integrated in the same order. To begin, consider the conventional panel ARDL specification presented in Equation 6, in which LEX is the dependent variable:


$${\mathrm{LEX}}_{i,t}=\sum_{j=1}^{p}\;\phi_{\mathrm{ij}}{\mathrm{LEX}}_{i,t-j}+\sum_{j=0}^{q}\;\gamma_{\mathrm{ij}}X_{i,t-j}+\alpha_{i}+\varepsilon_{i,t}$$ (6)

Here, ${\mathrm{LEX}}_{i,t-j}$ represents lagged values of the dependent variable (life expectancy), and $X_{i,t-j}$ denotes a vector of independent variables ( ${\mathrm{CO}}_{2}$ emissions, ICT, urbanization, institutional quality, and GDP). The term $\alpha_{i}$ captures individual fixed effects, $\phi_{\mathrm{ij}}$ and $\gamma_{\mathrm{ij}}$ are the coefficients of the lagged dependent and independent variables, respectively, and $\varepsilon_{i,t}$ is the error term. However, standard panel ARDL models like Equation 6 may yield biased results in the presence of CSD. To address this, we apply the CS-ARDL model, which corrects for CSD by incorporating cross-sectional averages of both dependent and explanatory variables, ensuring more robust and efficient estimation. Chudik and Pesaran (65) recommend augmenting the standard ARDL by adding lags of the cross-sectional averages. The modified CS-ARDL specification is presented in Equation 7:


$${\mathrm{LEX}}_{i,t}=\sum_{j=1}^{p}\;\phi_{\mathrm{ij}}{\mathrm{LEX}}_{i,t-j}+\sum_{j=0}^{q}\;X_{i,t-j}+\sum_{j=0}^{r}\;\beta_{\mathrm{ij}}^{\prime }{\bar{W}}_{t-j}+\alpha_{i}+\varepsilon_{i,t}$$ (7)

In Equation 7, ${\bar{W}}_{t-j}=({\bar{\mathrm{LEX}}}_{t-j},{\bar{X}}_{t-j})$ are the cross-sectional averages of the dependent and explanatory variables, respectively. These averages control for unobserved common factors and eliminate bias from CSD. The superscripts p,q , and r indicate the number of lags for the dependent variable, regressors, and their cross-sectional averages. The long-run coefficient for CS-ARDL can be derived using the following formula:


$${\overset{ˆ}{\theta }}_{\mathrm{CS}-\text{ARDL},\mathrm{ij}}=\frac{\sum_{j=0}^{q}\;\;{\overset{ˆ}{\gamma }}_{\mathrm{ij}}}{1-\sum_{j=1}^{p}\;\;{\overset{ˆ}{\phi }}_{\mathrm{ij}}}$$

Equation 7 can also be expressed in error correction form as follows:


$$\begin{array}{l} \Delta {\mathrm{LEX}}_{i,t}=\vartheta_{i}[{\mathrm{LEX}}_{i,t-1}-\lambda_{i}X_{i,t-1}]-\sum_{j=1}^{p-1}\phi_{\mathrm{ij}}\Delta {\mathrm{LEX}}_{i,t-j}\\ \;\;\;\;\;\;\;\;\;\;\;\;\;\;\;\;\;\;\;\;\;\;+\sum_{j=0}^{q}\gamma_{\mathrm{ij}}\Delta X_{i,t-j}+\sum_{j=0}^{r}\beta_{\mathrm{ij}}^{\prime }{\bar{W}}_{t-j}+\phi 9)+\varepsilon_{i,t} \end{array}$$ (8)

Where in Equation 8, Δ denotes the first difference operator and $\vartheta_{i}$ is the adjustment coefficient, measuring the speed at which deviations from the long-run equilibrium are corrected.

### 3.3 Data

**Figure 1:**
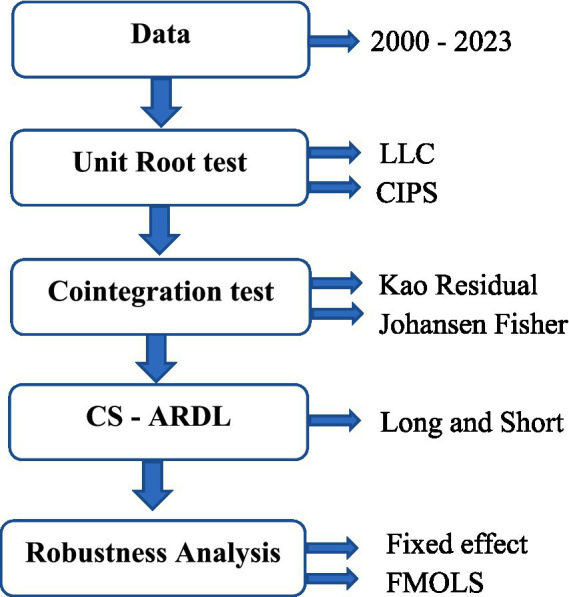
Estimation strategy.

This study examines six core variables: life expectancy (LEX), carbon dioxide emissions (CO_2_e) per capita, information and communication technology (ICT), urbanization (URB), institutional quality (IQ), and gross domestic product per capita (GDP). Data for LEX, CO_2_e, URB, ICT, and GDP were obtained from the World Development Indicators (WDI), whereas institutional quality indicators were collected from the Worldwide Governance Indicators (WGI). The variables are defined as follows: CO_2_e are measured in metric tons per capita; life expectancy is reported in total years at birth; urbanization is represented by the urban population as a percentage of the total; and GDP per capita is measured in constant 2015 U.S. dollars. Institutional quality index is calculating from six governance dimensions: X1: Control of Corruption, X2: Government Effectiveness, X3: Political Stability and Absence of Violence/Terrorism, X4: Regulatory Quality, X5: Rule of Law and X6: Voice and Accountability as suggested by Uddin et al. (62). Table A1 reports the PCA results for the institutional quality index. The first principal component has an eigenvalue of 4.738 and explains about 79 percent of the total variance, indicating that a single latent factor captures most of the common variation in institutional quality indicators. The cumulative variance explained by the first two components rises to 91.6 percent, while the remaining components contribute only marginally, suggesting limited additional explanatory power. The factor loadings show that X1, X2, X3, X4, X5 and X6 all load positively and strongly on the first component. This confirms that Comp1 represents a broad and comprehensive measure of institutional quality. Therefore, the first principal component is used to construct the institutional quality index in this study. The ICT index was constructed using four components as suggested by Verma et al. (63) and Zhang et al. (64), Fixed Broadband Subscriptions, Fixed Telephone Subscriptions, Mobile Cellular Subscriptions (per 100 people), and the percentage of individuals using the Internet. Table A2 presents the PCA results for the ICT index. The first principal component has an eigenvalue of 2.49 and explains 62.2 percent of the total variance, indicating that it captures the dominant common variation among the ICT indicators. When the second component is added, the cumulative explained variance increases to 86.7 percent, while the remaining components contribute only marginally to the overall variation. The factor loadings reveal that fixed broadband subscriptions (FBS), mobile cellular subscriptions (MCS), and individuals using the internet (IUI) load strongly and positively on the first component, whereas fixed telephone subscriptions (FTS) loads mainly on the second component. This suggests that Comp1 reflects overall ICT penetration and digital connectivity. Therefore, consistent with standard practice, the first principal component is used to construct the ICT index in this study. The estimation framework is illustrated in Figure 1.

## 4 Results and discussion

In Table 1, shows the descriptive statistics, the mean shows the average value, while the median represents the central value of each variable. For LEX, the mean (4.2335) and median (4.2524) are very close, indicating a fairly balanced distribution. CO_2_e shows a lower mean (1.5163) than the median (1.9013), suggesting some lower extreme values. ICT has a mean close to zero, reflecting overall low average intensity with some variation around the center. URB has similar mean (4.0493) and median (4.1538), implying stability in urbanization levels. For IQ, the median (0.6737) is higher than the mean (−2.00E−08), pointing to the presence of negative extreme values. GDP shows a slightly lower mean (8.5121) than the median (8.7226), indicating mild left-side dispersion. LEX has a very small standard deviation (0.0921), indicating high stability over time. CO_2_e (0.8454) and URB (0.3601) show moderate variability. ICT (1.5778) and IQ (2.1767) exhibit relatively high dispersion, reflecting substantial fluctuations. GDP shows moderate variation with a standard deviation of 0.7560. For all variables, the Jarque–Bera statistics are relatively high and the corresponding probabilities are below 0.05. This indicates that LEX, CO_2_e, ICT, URB, IQ, and GDP all reject the null hypothesis of normality, suggesting that their distributions deviate from a normal distribution. Table 2 shows the CSD test results, all four tests indicate significant CSD, as all *p*-values are 0.000. The Breusch-Pagan LM test (122.9245) strongly rejects the null of independence. Pesaran’s scaled LM and bias-corrected LM also confirm dependence with significant values. The Pesaran CD test (5.1675) further supports this result, especially for large panels.

**Table 1:** Descriptive statistics.

	LEX	CO_2_e	ICT	URB	IQ	GDP
Mean	4.2335	1.5163	7.50E−09	4.0493	−2.00E−08	8.5121
Median	4.2524	1.9013	−0.1764	4.1538	0.6737	8.7226
Maximum	4.3593	2.6665	3.2144	4.4749	2.8270	9.4322
Minimum	3.9874	−0.0633	−2.3610	3.3202	−5.5291	6.6290
Std. Dev.	0.0921	0.8454	1.5778	0.3601	2.1767	0.7560
Skewness	−0.9487	−0.3063	0.2703	−0.7156	−1.1701	−1.1305
Kurtosis	3.4070	1.5955	1.8612	2.1365	3.5387	3.0219
Jarque-Bera	18.8281	11.7395	7.9455	13.9693	28.8325	25.5652
Probability	0.0001	0.0028	0.0188	0.0009	0.0000	0.0000

**Table 2:** Cross sectional dependency.

Test	Statistic	Prob.
Breusch-Pagan LM	122.9245^*^	0.0000
Pesaran scaled LM	25.2507^*^	0.0000
Bias-corrected scaled LM	25.1420^*^	0.0000
Pesaran CD	5.1675^*^	0.0000

Note: *,** & *** represent the 1, 5% & 10% significance level.

Table 3 presents the results of the LLC and CIPS unit root tests. According to the LLC test, ICT and IQ are non-stationary at level, while LEX, CO_2_e, URB, and GDP are stationary. In contrast, the CIPS test shows that LEX and ICT are stationary at level, whereas CO_2_e, URB, IQ, and GDP are non-stationary. After applying the first difference, all variables become stationary under both tests, indicating they are integrated of order one, I(1). Therefore, the results confirm a mixed order of stationarity among the variables.

**Table 3:** Panel unit root test.

	LLC		CIPS	
	I(0)	I(1)	I(0)	I(1)
LEX	−3.383^*^	−4.051^*^	−3.080^*^	−7.743^*^
CO_2_e	−2.556^*^	−2.281^**^	−0.968	−10.643^*^
ICT	0.464	−4.532^*^	−2.286^**^	−3.863^*^
URB	−33.976^*^	−14.753^*^	−0.325	−2.954^*^
IQ	1.196	−1.7705^***^	−2.1533	−4.003^*^
GDP	−2.350^*^	−7.632^*^	−0.805	−5.532^*^

*,** & *** represent the 1, 5% & 10% significance level.

Table 4 presents the results of the Kao residual cointegration test. The t-statistic of −1.7241 with a p-value of 0.0423 is statistically significant at the 5% level, leading to the rejection of the null hypothesis of no cointegration. This indicates the existence of a long-run equilibrium relationship among the panel variables. The residual variance and HAC variance, both at 0.0002 and 0.0002, suggest low residual dispersion and stable estimates. Table 5 reports the Johansen Fisher panel cointegration test results. Both the Trace and Max-Eigen statistics confirm the presence of six cointegrating equations, further supporting the existence of a stable long-run relationship among the variables.

**Table 4:** Kao residual cointegration test.

	t-Statistic	Prob.
ADF	−1.7241^**^	0.0423
Residual variance	0.0002	
HAC variance	0.0002	

Note: *,** & *** represent the 1, 5% & 10% significance level.

**Table 5:** Johansen Fisher panel cointegration test.

No. of CE(s)	Trace test	Prob.	Max-eigen test	Prob.
None	289.9000^*^	0.0000	157.9000^*^	0.0000
At most 1	194.0000^*^	0.0000	114.7000^*^	0.0000
At most 2	117.0000^*^	0.0000	64.1600^*^	0.0000
At most 3	66.9700^*^	0.0000	43.5500^*^	0.0000
At most 4	35.9800^*^	0.0001	23.8300^*^	0.0081
At most 5	31.0900^*^	0.0006	31.0900^*^	0.0006

Note: *,** & *** represent the 1, 5% & 10% significance level.

Table 6 presents the CS–ARDL estimates. In both the long run and the short run, ICT, URB, IQ, and GDP exert a positive effect on LEX, whereas CO_2_e have an adverse effect on LEX. The coefficient of CO_2_e is negative, indicating that a 1% increase in CO_2_e leads to a 0.1152% decrease in LEX. These findings are consistent with those of Osabohien et al. (14), Das and Debnath (9), Azam and Adeleye (35), and Guo et al. (38). Osabohien et al. (14) examined the impact of energy consumption and CO_2_e on LEX in Nigeria and found that CO_2_e significantly reduces LEX. Das and Debnath (9) investigated the net impact of CO_2_e on LEX in India and explored whether the country has exceeded its optimal CO_2_e threshold. Their results confirmed a long-run quadratic relationship, showing that CO_2_e reduces LEX. Azam and Adeleye (35) analyzed the effects of CO_2_e from liquid and solid fuels on LEX in the Asia-Pacific region and found that both fuel types negatively affect LEX, with liquid fuels having the strongest impact. Guo et al. (38) assessed the effects of CO_2_e, urbanization, and renewable energy on LEX and infant mortality in SAARC countries, concluding that CO_2_e reduce LEX and increase infant mortality, while renewable energy and urbanization improve health outcomes.

**Table 6:** CS – ARDL estimates.

Variable	Long run			Short run		
	Coefficient	Std. Err.	Prob.	Coefficient	Std. Err.	Prob.
CO_2_e	−0.1152^*^	0.0405	0.0000	−0.2198^*^	0.0542	0.0000
ICT	0.0950^*^	0.0155	0.0000	0.0234^**^	0.0112	0.0497
URB	0.0532^**^	0.0260	0.0486	0.6383^*^	0.0360	0.0000
IQ	0.0579^*^	0.0080	0.0000	0.0425^*^	0.0060	0.0000
GDP	0.5066^*^	0.1337	0.0003	0.2581^*^	0.0487	0.0000
ECM(−1)	–	–	–	−0.1480^**^	0.0708	0.0302

Note: *,** & *** represent the 1, 5% & 10% significance level.

The coefficient of ICT is 0.0950, indicating that a one-unit increase in ICT leads to a 0.0950% increase in LEX. The positive sign of ICT reflects its role in promoting LEX by improving access to healthcare information and services. Moreover, ICT supports health education and awareness, encouraging healthier lifestyles and preventive care. It also strengthens healthcare systems by improving data management, coordination, and service delivery efficiency. These results are consistent with the findings of Majeed and Khan (41), Alhassan and Adam (44), Ronaghi (40), and Bayar et al. (42). Majeed and Khan (41) examined the contribution of ICT infrastructure to health outcomes in 184 countries during 1990–2014 and found that internet usage, mobile subscriptions, and fixed telephone lines significantly improved LEX. Alhassan and Adam (44) analyzed the effects of digital inclusion and ICT access on quality of life at the global level, reporting significant improvements. Ronaghi (40) investigated the impact of ICT on LEX in Middle Eastern countries and found a significant positive effect. Bayar et al. (42) explored the causal relationship between ICT indicators and LEX in emerging market economies and concluded that ICT particularly mobile and internet usage significantly enhances LEX. Similarly, Shao et al. (22) identified ICT as a key mediator of national health outcomes across 141 countries. Zhang et al. (64) examined the impact of ICT, health expenditure and IQ on LEX in China, and they found that ICT, health expenditure and IQ have positive effect on LEX.

The coefficient of URB is 0.0532, indicating that a 1% increase in urbanization increases LEX by 0.0532%. Urbanization can improve LEX by enhancing access to healthcare facilities, clean water, and sanitation services. Urban areas typically offer better infrastructure, education, and public health programs, which support healthier lifestyles. They also provide more employment opportunities and higher incomes, improving living standards and access to adequate nutrition. These findings are consistent with Zhang et al. (30) and Guo et al. (38), but contrast with Erum et al. (48). Zhang et al. (30) found that urbanization, education, and green growth positively influence LEX, although CO_2_e exerts a negative effect. Guo et al. (38), focusing on SAARC countries, reported that urbanization, renewable energy, GDP, and industrialization increase LEX, while CO_2_e consistently reduces it. In contrast, Erum et al. (48) examined highly polluted countries from 1990 to 2022 and found that poor air quality and urbanization reduce LEX, while inclusive development and good governance improve it. Their results also showed that urbanization can exacerbate pollution-related health damage.

In the context of BRICS economies, URB and digitalization exhibit heterogeneous effects on LEX. URB can enhance health outcomes by improving access to healthcare facilities, sanitation, education, and employment opportunities, thereby contributing positively to LEX. Concurrently, digitalization through expanded internet access, mobile connectivity, and e-health services facilitates health information dissemination, telemedicine, and more efficient public service delivery, further supporting longevity. However, rapid and unplanned urban expansion often exacerbates environmental degradation, air pollution, congestion, and social inequality, which may offset these gains. Likewise, digital divides across income groups and regions can limit the health benefits of technological advancement. Consequently, the net effect of urbanization and digitalization on LEX in BRICS countries depends critically on environmental governance, institutional quality, and inclusive development strategies.

The coefficients of IQ and GDP are 0.0579 and 0.5066, respectively. This implies that a one-unit increase in institutional quality raises LEX by 0.0579%, while a 1% increase in GDP increases LEX by 0.5066%. Effective institutions support hospital management, enforce health regulations, and reduce corruption, allowing economic growth to translate into tangible health improvements. In contrast, weaker institutional frameworks in parts of India, Russia, and South Africa may constrain the health benefits of economic growth. This indicates that in BRICS countries, LEX improves when economic resources are complemented by strong institutional structures that ensure effective healthcare delivery. These results are consistent with Ibrahim and Ajide (51), Uddin et al. (52), and Hadipour et al. (53). Ibrahim and Ajide (51) studied the role of income levels and institutional quality in four oil-producing African countries and found that income mitigates negative effects, while institutional quality influences outcomes. Uddin et al. (52) revisited the determinants of LEX in SAARC economies and confirmed a positive effect of institutional quality. Hadipour et al. (53), using data from 158 economies, found that institutional quality, GDP, schooling, and urbanization improve LEX, whereas CO_2_e reduces it. Zhang et al. (30) further showed that the rule of law positively affects LEX by improving equality, reducing corruption, ensuring efficient resource allocation, and enforcing health, safety, and environmental standards.

Finally, the short-run CS–ARDL results confirm that CO_2_e negatively affects LEX, while digitalization, urbanization, and institutional quality exert positive effects. The error correction term, ECM(−1), has a coefficient of −0.1480 and is statistically significant, indicating a stable long-run relationship. This implies that approximately 14.80% of short-run disequilibrium in LEX is corrected in each period, meaning that deviations from the long-run equilibrium gradually adjust back over time.

In this study, we used Fixed Effects and AMG estimations as robustness checks for the CS-ARDL analysis (see Table 7). Both methods confirm the statistical significance of all variables in explaining LEX. ICT, urbanization, GDP, and IQ show a positive relationship with LEX in both models. CO_2_ emissions exhibit a negative association, indicating their adverse impact. The consistency in the direction of coefficients across both methods supports the reliability of the findings. Differences in magnitudes reflect the short-run focus of Fixed Effects and the long-run nature of AMG. Finally, the robustness analysis strengthens the validity of the estimated relationships.

**Table 7:** Robustness analysis.

Variable	Fixed effect			AMG		
	Coefficient	Std. Error	Prob.	Coefficient	Std. Err.	Prob.
CO_2_e	−0.0670^**^	0.0310	0.0330	−0.3846^*^	0.0653	0.0000
ICT	0.0090^**^	0.0040	0.0270	0.0891^*^	0.0218	0.0001
URB	0.7180^*^	0.0700	0.0000	1.1614^*^	0.2736	0.0000
IQ	0.1220^*^	0.0160	0.0000	0.0432^**^	0.0188	0.0234
GDP	0.1270^*^	0.0430	0.0040	1.1185^*^	0.1369	0.0000

Note: *,** & *** represent the 1, 5% & 10% significance level.

## 5 Conclusion and policy recommendations

This study investigates the impact of CO_2_e, digitalization, urbanization, and institutional quality on LEX in the BRICS economies over the period 2000–2023. To capture both short-run and long-run dynamics, the analysis employs the CS-ARDL model, while Fixed Effects and Fully FMOLS estimators are used to ensure the robustness of the results. The long-run and short-run estimates from the CS-ARDL model reveal that CO_2_e exerts a negative and statistically significant effect on LEX, indicating that higher emissions are associated with a decline in population health and longevity. In contrast, digitalization (ICT), urbanization, IQ, and GDP all show positive and significant effects on LEX, suggesting that technological advancement, improved urban infrastructure, strong institutions, and higher economic performance contribute to better health outcomes and longer lives. These findings are consistent across robustness checks performed using the Fixed Effects and FMOLS estimators, both of which confirm the direction and significance of the relationships identified in the CS-ARDL model. The convergence of results across multiple estimation techniques enhances the credibility of the conclusion that environmental, technological, and institutional factors play a critical role in shaping life expectancy in the BRICS countries.

This study has some practical policy, that how each BRICS country can follow or learn from the others in implementing policies to improve life expectancy through air pollution control, digitalization, urbanization, and institutional quality: First, Brazil’s strong environmental regulations and urban green projects can serve as a model for other BRICS countries, such as India and China, to expand green spaces and enforce stricter emission standards. Second, China’s large-scale investment in renewable energy to reduce coal dependence provides an example for India, South Africa, and Russia to shift toward cleaner energy sources. Third, India can adopt China and Brazil’s approaches in monitoring and penalizing polluters effectively to control air pollution. Fourth, China’s advanced telemedicine and digital health platforms can guide other BRICS countries, particularly India and Brazil, in improving rural healthcare access. Fifth, India can follow China in integrating national-level electronic health records to efficiently track patient health. Sixth, Brazil can learn from China and India in promoting digital health literacy campaigns to increase citizen engagement with health technologies. Seventh, China’s sustainable urban infrastructure and planned city expansion offer lessons for Russia and India to prevent overcrowding and improve healthcare access. Eighth, Brazil’s focus on building hospitals and clinics in urban centers can help other BRICS countries strengthen healthcare services in growing cities. Ninth, South Africa can adopt urban planning policies from China and Brazil to balance rapid urban growth with healthier living environments. Tenth, Brazil’s experience in enforcing public health programs through relatively strong governance can guide India and South Africa in improving accountability in healthcare delivery. Eleventh, China’s effective regulatory frameworks demonstrate how economic resources can be translated into health improvements, offering lessons for other BRICS nations. Twelfth, Russia can follow Brazil and China in supporting evidence-based policymaking by using reliable health and environmental data to design impactful interventions.

This study also recommended some general policy implications for policymakers and governments to promote longer lives in BRICS economies. First, governments should implement strict regulations and carbon pricing mechanisms (e.g., carbon taxes or cap-and-trade systems) to reduce industrial CO_2_ emissions. Second, promote renewable energy (solar, wind, hydro) through subsidies and green financing. Cleaner energy reduces environmental toxins, leading to healthier ecosystems and reduced disease burden. Third, the government should expand digital infrastructure in underserved regions. It increases access to telehealth, emergency alerts, and online health services, particularly in remote or rural areas. Fourth, integrate ICT into healthcare systems (e-health, telemedicine, mobile health apps) to enhance access to medical services. Fifth, support digital literacy programs, especially for older adults and healthcare workers, to ensure effective utilization of health technologies. Sixth, develop innovative city policies that prioritize clean water, sanitation, green spaces, and pollution control to create healthier urban environments. Seventh, invest in affordable and accessible healthcare, housing, and transport services in urban areas to reduce inequality and improve quality of life. Eight, implement urban planning that limits overcrowding and promotes mixed land use to ensure balanced development and public health outcomes. Ninth, establish independent health oversight bodies and implement strict anti-corruption laws. This reduces misallocation of resources, provides better delivery of healthcare services, and improves access to essential medicines, leading to better health outcomes and longer life spans. Tenth, ensures clean air, safe water, and hygienic living conditions—reducing exposure to diseases and chronic health risks. Eleventh, guarantee legal access to healthcare as a fundamental right and establish grievance redressal mechanisms for public health complaints. Twelfth, protects vulnerable populations, ensures timely medical care, and holds providers accountable, thereby preventing premature deaths and improving overall population health. By implementing these targeted policies, BRICS countries can directly address the determinants of life expectancy through environmental protection, digital innovation, urban wellbeing, institutional effectiveness, and improved health outcomes.

This study has several limitations that provide avenues for future research. First, air pollution was measured using only CO_2_ emissions as an indicator. Future studies should incorporate additional pollutants, such as PM2.5 and NO_2_, to capture a more comprehensive picture of environmental impacts on life expectancy. Second, the study does not fully address endogeneity concerns. The CS-ARDL model cannot account for potential bidirectional relationships between variables, such as the reciprocal effects of life expectancy and economic growth, nor does it apply techniques like the instrumental variable approach to correct for this issue. Third, the analysis does not examine interaction effects between key variables. For instance, it overlooks potential interactions between urbanization and air pollution, as well as the moderating role of institutional quality in shaping the health impacts of digitalization. As a result, the study cannot explain the sources of heterogeneity in the effects of these variables, leaving important questions about the underlying mechanisms unanswered. Future studies should address these gaps by incorporating multiple air pollution indicators, applying advanced econometric techniques to handle endogeneity, and examining interaction effects between key variables. This approach would provide a deeper understanding of how factors such as urbanization, digitalization, and institutional quality jointly influence life expectancy and help identify the conditions under which their effects vary across different BRICS economies.

## Data Availability

The raw data supporting the conclusions of this article will be made available by the authors without undue reservation.

## Appendix

**Table A1:** PCA output for intuitional quality index.

Component	Eigenvalue	Difference	Proportion	Cumulative		
Comp1	4.738	3.98	0.79	0.79		
Comp2	0.758	0.485	0.126	0.916		
Comp3	0.273	0.136	0.045	0.962		
Comp4	0.137	0.078	0.023	0.984		
Comp5	0.059	0.025	0.01	0.994		
Comp6	0.034	–	0.006	1		
Principal components (eigenvectors)						
Variable	Comp1	Comp2	Comp3	Comp4	Comp5	Comp6
X1	0.438	−0.056	−0.055	−0.705	−0.547	−0.066
X2	0.424	−0.306	−0.452	0.225	0.043	0.686
X3	0.398	−0.363	0.7	−0.13	0.445	0.068
X4	0.439	−0.044	0.178	0.655	−0.463	−0.36
X5	0.435	0.142	−0.474	−0.068	0.532	−0.528
X6	0.296	0.866	0.218	0.024	0.056	0.335

**Table A2:** PCA output for ICT index.

Component	Eigenvalue	Difference	Proportion	Cumulative
Comp1	2.49	1.512	0.622	0.622
Comp2	0.977	0.532	0.244	0.867
Comp3	0.445	0.356	0.111	0.978
Comp4	0.089	–	0.022	1
Principal components (eigenvectors)				
Variable	Comp1	Comp2	Comp3	Comp4
FBS	0.539	0.082	−0.766	0.342
FTS	0.178	0.961	0.203	−0.057
MCS	0.557	−0.2	0.607	0.531
IUI	0.607	−0.171	0.063	−0.774
